# Novel variants require established frameworks: emphasizing the role of ISTH diagnostic and classification guidelines in congenital fibrinogen disorders

**DOI:** 10.1186/s41065-025-00435-2

**Published:** 2025-04-22

**Authors:** Mustafa Vakur Bor

**Affiliations:** 1 Unit for Thrombosis Research, Department of Regional Health Science, University of Southern Denmark, Esbjerg,, Denmark; 2 Department of Clinical Biochemistry, University Hospital of Southern Denmark, Finsensgade 35, Esbjerg, 6700 Denmark

## Abstract

This commentary aims to highlight the importance of applying the diagnostic and classification guidelines of the International Society on Thrombosis and Haemostasis (ISTH), along with standardized bleeding assessment tools, in the evaluation of patients with congenital fibrinogen disorders. Additionally, it addresses key laboratory methodologies relevant to the diagnosis of these conditions. We believe that this commentary will contribute meaningfully to the ongoing discussions and promote the adoption of standardized approaches in the assessment of rare congenital fibrinogen disorders.

I read with great interest the article by Xie et al., titled *“A novel mutation in the Fibrinogen gamma-chain ( FGG) causes hypofibrinogenemia in a Chinese family”*, published in *Hereditas* [1]. The authors describe a three-generation family-four members with hypofibrinogenemia caused by a novel pathogenic variant in the FGG gene (c.668G > C, p.Arg223Thr). The authors further explore genotype–phenotype correlations by analyzing fibrin structure in affected individuals using electron microscopy. Notably, the authors suggest that both fibrinogen deficiency and clinical severity were more pronounced in the third generation compared to the second [1].

While this study contributes valuable insights and introduces a previously unreported fibrinogen variant, several important considerations merit further discussion. This commentary seeks to address these points in the context of current guidelines and existing evidence.

Congenital fibrinogen disorders (CFDs) represent a heterogeneous group of rare inherited coagulopathies, including hypofibrinogenemia, afibrinogenemia, dysfibrinogenemia, and hypodysfibrinogenemia, that collectively account for approximately 8% of all rare bleeding disorders [2, 3]. Due to their clinical complexity, the International Society on Thrombosis and Haemostasis (ISTH), along with its Scientific and Standardization Committee (SSC) on Factor XIII and Fibrinogen, discusses issues relevant to the diagnostic approach to CFD and recommends a classification system based on both fibrinogen levels and clinical phenotype [3]. Accurate classification is essential for appropriate diagnosis and clinical management. However, this classification guideline was not applied to the patients in the study by Xie et al. [1]. Additionally, no standardized bleeding assessment—such as the ISTH Bleeding Assessment Tool [4] or a validated bleeding score for rare bleeding disorders [5] —was employed, thereby limiting the clinical interpretation of the findings [1].

As established in the literature [2, 6, 7] and also noted by the authors [1], individuals with Fg: C > 1.0 g/L are generally asymptomatic, whereas levels < 0.7 g/L are associated with spontaneous or trauma-induced bleeding. In this study, patients II:1 and II:4, with Fg: C > 1.0 g/L, had mild hypofibrinogenemia and would be classified as Type 2 C under the ISTH classification [3]. Notably, patient II:4 underwent two cesarean sections without bleeding or thrombotic complications. Patients III:1 and III:3, with Fg: C < 1.0 g/L, had moderate hypofibrinogenemia and would be classified as Type 2B [3]. Although the authors did not report spontaneous bleeding, self-reported occasional bruising of the skin and prolonged bleeding following trauma were described. However, in the absence of standardized bleeding scores, the clinical phenotype from this study remains incompletely characterized. The authors propose that disease severity increases across generations based on fibrinogen levels and clinical presentation. This interpretation is not supported by objective bleeding assessments; as all patients would be classified as likely asymptomatic according to ISTH Bleeding Assessment Tool [4].

The use of fibrinogen activity (Clauss) and antigenic assays is appropriate and aligns with ISTH diagnostic guideline for CFD [3]. However, the rationale for including the PT-derived fibrinogen assay, an indirect and less specific approach [3, 8], is not clearly justified. Although thromboelastography confirmed low fibrinogen activity, these additional tests did not appear to contribute further diagnostic value. Turbidimetric analysis of fibrin polymerization, as a complementary method to electron microscopy [7], could have further strengthened the evaluation of the genotype–phenotype correlation associated with the novel variant.

Finally, as noted by the authors, fibrinogen levels were interestingly lower in the third generation despite the presence of the same FGG variant. Although no explanation is provided, additional fibrinogen polymorphisms may contribute to this discrepancy and clinical phenotype [9, 10]. Given that whole-exome sequencing was performed, it can be assumed that such possibilities were considered, although this is not explicitly stated.

In conclusion, while this study offers novel insights into a novel fibrinogen variant, its clinical and diagnostic value would be strengthened by adherence to established ISTH diagnostic and classification guideline for CFDs, use of standardized bleeding assessment tools, clarification of methodological choices, and further exploration of potential genetic modifiers.

## Data Availability

No datasets were generated or analysed during the current study.
